# Supplementary material for: Anakinra in hospitalized COVID-19 patients guided by baseline soluble urokinase plasminogen receptor plasma levels: A real world, retrospective cohort study
Source: PLoS One. 2023 Apr 4;18(4):e0273202. doi: 10.1371/journal.pone.0273202 (PMC10072376; doi:10.1371/journal.pone.0273202)

**S1 Figure 1.** Covariates balance after applying the generalized boosted model to estimate the propensity score of receiving anakinra


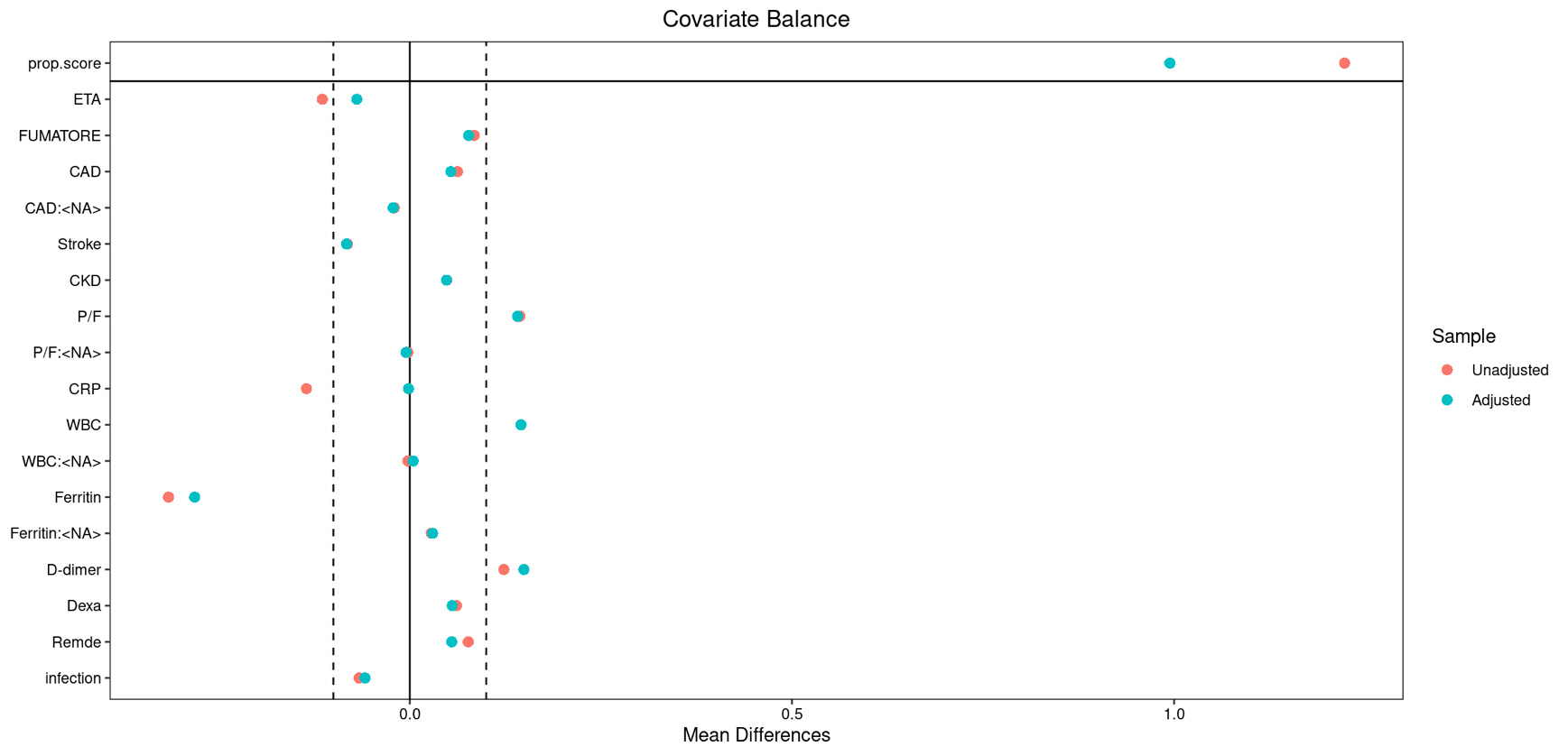

Supplement: S1 Fig — (DOCX) [file pone.0273202.s001.docx]
