# Supplementary material for: Anakinra in hospitalized COVID-19 patients guided by baseline soluble urokinase plasminogen receptor plasma levels: A real world, retrospective cohort study
Source: PLoS One. 2023 Apr 4;18(4):e0273202. doi: 10.1371/journal.pone.0273202 (PMC10072376; doi:10.1371/journal.pone.0273202)
Supplement: S1 Table — (DOCX) [file pone.0273202.s002.docx]

# **S1 Table. Patients with suPAR < 6 ng/mL vs patients treated with anakinra**

| **Characteristic** | **Overall**, N = 104*^1^* | **Control Group 2^a^** | **Anakinra Group** | ***p*-value^b^** |  |
| --- | --- | --- | --- | --- | --- |
|  |  |  |  |  |  |
| Demographic | | | | |  |
| Age (years) | 65 (53, 75) | 63 (50, 74) | 67 (57, 78) | 0.11 |  |
| Male sex | 67/104 (64) | 28/48 (58) | 39/56 (70) | 0.23 |  |
| Vaccination | 42/100 (42) | 20/45 (44) | 22/55 (40) | 0.65 |  |
| BMI | 26.6 (24.1, 30.4) | 27.2 (22.6, 31.7) | 26.2 (24.7, 28.7) | 0.69 |  |
| Comorbidities | | | | |  |
| Tabagism | 10/104 (9.6) | 3/48 (6.2) | 7/56 (12) | 0.33 |  |
| COPD | 9/104 (8.7) | 4/48 (8.3) | 5/56 (8.9) | >0.99 |  |
| HBP | 48/104 (46) | 21/48 (44) | 27/56 (48) | 0.65 |  |
| CAD | 15/104 (14) | 1/48 (2.1) | 14/56 (25) | <0.001 |  |
| CHF | 4/104 (3.8) | 1/48 (2.1) | 3/56 (5.4) | 0.62 |  |
| AF | 8/104 (7.7) | 2/48 (4.2) | 6/56 (11) | 0.28 |  |
| CVD | 3/104 (2.9) | 3/48 (6.2) | 0/56 (0) | 0.095 |  |
| DM | 18/104 (17) | 6/48 (12) | 12/56 (21) | 0.30 |  |
| CKD | 8/104 (7.7) | 3/48 (6.2) | 5/56 (8.9) | 0.72 |  |
| Other information | | | | |  |
| Symptoms onset - admission | 7.0 (4.0, 9.0) | 6.5 (5.0, 9.0) | 7.0 (3.8, 9.0) | 0.62 |  |
| Bacterial co-infection | 2/104 (1.9) | 1/48 (2.1) | 1/56 (1.8) | >0.99 |  |
| Bacterial sovra-infection | 14/104 (13) | 5/48 (10) | 9/56 (16) | 0.40 |  |
| SCOPE score | 8.00 (6.00, 9.00) | 6.00 (5.00, 8.00) | 8.00 (6.25, 9.00) | 0.006 |  |
| Laboratory | | | | |  |
| suPAR (ng/mL) | 5.95 (4.80, 8.10) | 4.80 (4.10, 5.30) | 8.00 (6.80, 9.75) | <0.001 |  |
| PaO2/FiO2 ratio | 252 (212, 285) | 274 (241, 295) | 240 (198, 272) | 0.004 |  |
| Lymphocytes (μl) | 1,030 (760, 1,315) | 1,165 (880, 1,480) | 910 (730, 1,200) | 0.067 |  |
| WBCs (μl) | 6,210 (4,695, 8,405) | 6,335 (4,195, 9,612) | 6,200 (5,005, 8,110) | 0.88 |  |
| IL-6 (ng/L) | 17 (8, 44) | 13 (7, 29) | 32 (8, 60) | 0.049 |  |
| Ferritin (ng/mL) | 529 (251, 1,182) | 530 (253, 1,080) | 515 (246, 1,321) | 0.48 |  |
| D-dimer (ng/mL) | 711 (530, 1,293) | 580 (367, 1,293) | 790 (575, 1,300) | 0.032 |  |
| CRP (mg/L) | 64 (36, 108) | 60 (20, 94) | 68 (39, 124) | 0.034 |  |
| In-hospital therapy | | | | |  |
| Dexamethasone | 97/103 (94) | 41/47 (87) | 56/56 (100) | 0.008 |  |
| Remdesivir | 36/103 (35) | 19/47 (40) | 17/56 (30) | 0.41 |  |
| Tocilizumab | 2/103 (1.9) | 2/47 (4.3) | 0/56 (0) | 0.21 |  |
| Tocilizumab 2 doses | 2/104 (1.9) | 2/48 (4.2) | 0/56 (0) | 0.21 |  |
| Monoclonal antibodies | 7/103 (6.8) | 6/47 (13) | 1/56 (1.8) | 0.045 |  |
| Outcomes | | | | |  |
| Supplementary oxygen (days) | 9 (6, 14) | 7 (5, 11) | 10 (8, 15) | 0.003 |  |
| Lenght of stay (days) | 11 (8, 16) | 10 (7, 14) | 11 (8, 18) | 0.033 |  |
| HFNC | 22/104 (21) | 6/48 (12) | 16/56 (29) | 0.045 |  |
| PaO2/FiO2 < 100 | 8/104 (7.7) | 1/48 (2.1) | 7/56 (12) | 0.066 |  |
| PaO2/FiO2 < 150 | 17/104 (16) | 4/48 (8.3) | 13/56 (23) | 0.041 |  |
| ICU | 2/104 (1.9) | 0/48 (0) | 2/56 (3.6) | 0.50 |  |
| NIV | 1/104 (1.0) | 0/48 (0) | 1/56 (1.8) | >0.99 |  |
| MV | 1/104 (1.0) | 0/48 (0) | 1/56 (1.8) | >0.99 |  |
| WHO Clinical Progression Scale |  |  |  | 0.39 |  |
| 1 | 70/100 (70) | 36/47 (77) | 34/53 (64) |  |  |
| 2 | 25/100 (25) | 9/47 (19) | 16/53 (30) |  |  |
| 3 | 4/100 (4.0) | 2/47 (4.3) | 2/53 (3.8) |  |  |
| 4 | 1/100 (1.0) | 0/47 (0) | 1/53 (1.9) |  |  |
| Death | 5/104 (4.8) | 1/48 (2.1) | 4/56 (7.1) | 0.37 |  |

^a^ patients admitted for COVID-19 pneumonia but who presented with baseline suPAR < 6 ng/mL

^b^ Wilcoxon rank sum test; Pearson's Chi-squared test
