# Supplementary material for: Anakinra in hospitalized COVID-19 patients guided by baseline soluble urokinase plasminogen receptor plasma levels: A real world, retrospective cohort study
Source: PLoS One. 2023 Apr 4;18(4):e0273202. doi: 10.1371/journal.pone.0273202 (PMC10072376; doi:10.1371/journal.pone.0273202)
Supplement: S2 Table — (DOCX) [file pone.0273202.s003.docx]

**S2 Table 2. Fully vaccinated vs non vaccinated individuals, descriptive statistics**.

|  | **Fully vaccinated** | **Non-vaccinated** |
| --- | --- | --- |
| Age, median (IQR) | 73 (64-81) | 63 (51-72) |
| BMI, median (IQR) | 27 (24.6-30) | 26 (24-30) |
| Comorbilities, freq (%) |  |  |
| Smoker | 8 (13) | 4 (5) |
| COPD | 10 (16) | 2 (2) |
| High blood pressure | 39 (63) | 32 (39) |
| Coronary artery disease | 19 (31) | 5 (6) |
| Congestive heart failure | 4 (6) | 3 (4) |
| Atrial fibrillation | 4 (6) | 4 (5) |
| Stroke | 3 (5) | 4 (5) |
| Diabetes mellitus | 20 (32) | 9 (11) |
| Chronic kidney disease | 9 (15) | 1 (1) |
| Laboratory values at baseline, median (IQR) |  |  |
| suPAR | 6,8 (4.9-10.5) | 7,4 (5.5-9.1) |
| SCOPE score | 7 (5-9) | 8 (7-9) |
| Baseline P/F | 261 (235-286) | 237 (199-279) |
| CRP, mg/L | 66,9 (32-127,4) | 86,5 (37-111) |
| IL-6, ng/L | 12,3 (5.4-43) | 25,3 (9-46) |
| Ferritin, ng/mL | 490 (163-1065) | 1017,0 (294-1509) |
| D-dimer, ng/mL | 870 (500-1901) | 745 (559-1043) |

COPD: chronic obstructive pulmonary disease;
