# Supplementary material for: Anakinra in hospitalized COVID-19 patients guided by baseline soluble urokinase plasminogen receptor plasma levels: A real world, retrospective cohort study
Source: PLoS One. 2023 Apr 4;18(4):e0273202. doi: 10.1371/journal.pone.0273202 (PMC10072376; doi:10.1371/journal.pone.0273202)
Supplement: S3 Table — (DOCX) [file pone.0273202.s004.docx]

**S3 Table**. Standardized mean differences (SMD) comparing patients with suPAR≥ 6 ng/mL treated with anakinra (1) and not treated with anakinra (0)

Stratified by Anakinra

0 1 SMD

n 49 56

Male = 1 (%) 32 (65.3) 39 ( 69.6) 0.093

Age (mean (SD)) 69.04 (14.45) 67.38 (14.65) 0.114

Smoker = 1 (%) 2 ( 4.1) 7 ( 12.5) 0.309

COPD = 1 (%) 4 ( 8.2) 5 ( 8.9) 0.027

HBP = 1 (%) 24 (49.0) 27 ( 48.2) 0.015

CAD = 1 (%) 9 (18.8) 14 ( 25.0) 0.152

CHF = 1 (%) 3 ( 6.1) 3 ( 5.4) 0.033

A. Fibrillation = 1 (%) 4 ( 8.2) 6 ( 10.7) 0.087

Stroke = 1 (%) 4 ( 8.2) 0 ( 0.0) 0.422

DM = 1 (%) 10 (20.4) 13 ( 23.2) 0.068

BMI (mean (SD)) 27.32 (4.66) 27.46 (5.57) 0.025

Vaccination = 1 (%) 20 (44.4) 22 ( 40.0) 0.090

P/F (mean (SD)) 228.79 (58.05) 236.93 (54.93) 0.144

CRP (mean (SD)) 108.62 (77.18) 95.59 (112.31) 0.135

Lymphocytes (mean (SD)) 1236.77 (965.98) 1161.49 (853.62) 0.083

WBC (mean (SD)) 6968.96 (3072.39) 8129.45 (10852.40) 0.146

IL-6 (mean (SD)) 46.83 (63.87) 47.99 (70.68) 0.017

Ferritin (mean (SD)) 1132.46 (1116.84) 832.12 (750.30) 0.316

D-dimer (mean (SD)) 2012.00 (3290.16) 2659.95 (6681.33) 0.123

Remdesivir = 1 (%) 12 (24.5) 18 ( 32.1) 0.170

coinfection = 1 (%) 12 (24.5) 10 ( 17.9) 0.163

Dexamethasone = 1 (%) 46 (93.9) 56 (100.0) 0.361
