# Supplementary material for: Anakinra in hospitalized COVID-19 patients guided by baseline soluble urokinase plasminogen receptor plasma levels: A real world, retrospective cohort study
Source: PLoS One. 2023 Apr 4;18(4):e0273202. doi: 10.1371/journal.pone.0273202 (PMC10072376; doi:10.1371/journal.pone.0273202)
Supplement: Attachment — Submitted filename: Response letter.docx [file pone.0273202.s006.docx]

Dear PLOS-One Editor

please find attached a point-by-point response to raised issues

*Thank you for your response to our query. We understand that this study is described as a retrospective study. However, how the anakinra/control groups were determined does suggest that there was some allocation to either a treatment or control group by the attending physicians (“The first control group (CG1) consisted of patients who retrospectively fulfilled the prescriptive criteria for anakinra but did not receive it at random at the discretion of the attending physicians”). There reporting here is not clear to us, or if this means that there were specific clinical reasons why the CG1 patients didn’t receive the treatment despite having higher suPAR levels; however, as written it looks as though some sort of assignment was made to a treatment. Although the accessing of the data could be retrospective, it is possible from the current methodology that a prospective assignment of treatments were done, which would then fulfil our criteria for a trial as there is a clear intervention (anakinra) and health outcome (length of hospital stay). Evidence for this is in the statement that “written informed consent for off-label use of anakinra was obtained to all individuals who received the study drug", suggesting that it was a prospective research study. In addition, although it is an emergency treatment, the patients were already on a cocktail of treatments as “standard of care” and therefore they are being given an experimental treatment."*

R: Thank you for the observation. Allocation to either Anakinra Group or CG1 was done at discretion of the attending physician, without real randomization or experimental purpose. It depended on the clinician individual expertise, in terms of both familiarity with the interpretation of suPAR levels and confidence with anakinra treatment for COVID-19. As mentioned in the discussion, the explanation for this heterogeneous approach it should be found on the fact that part of the medical team working in the study centre took part to the SAVE-MORE trial, and gained the necessary confidence to keep prescribing anakinra also in their daily practice, as an off-label medication. In addition, patients with COVID were admitted to different wards with

different medical teams, in 3 Infectious Diseases wards, in Internal Medicine and in Pulmonology. However, at the time of prescription, there was no intent to allocate patients to any “intervention” or “control” group. The Written informed consent for administration of any off-label medication was required by our institution. There were no specific clinical reasons why CG1 patients did not receive the study drug, and data collection, matching and group allocation were entirely retrospective. Thus, anakinra was prescribed only by experienced clinicians with no experimental intent, and patients who did not receive anakinra despite retrospectively fulfilling the prescriptive criteria (CG1) had the same characteristics of patients who did receive it.

In the manuscript, we added more information in the methods (lines 100-106), and discussion (lines 288-299) sections. Also, the word “random” has been removed from the manuscript, that we acknowledge it might sound misleading.

Kind regards,

Francesco Vladimiro Segala
