# Supplementary material for: Anakinra in hospitalized COVID-19 patients guided by baseline soluble urokinase plasminogen receptor plasma levels: A real world, retrospective cohort study
Source: PLoS One. 2023 Apr 4;18(4):e0273202. doi: 10.1371/journal.pone.0273202 (PMC10072376; doi:10.1371/journal.pone.0273202)
Supplement: Attachment — Submitted filename: Response to Rewievers.docx [file pone.0273202.s007.docx]

Dear PLOS-One Editor,

please find attached a point-by-point response to Journal Requirements and to Reviewers.

**Response to Journal Requirements**

1. *Please ensure that your manuscript meets PLOS ONE's style requirements, including those for file naming.*

R: Style requirements have been checked on the provided links. Supplementary information have been renamed and title page has been reformatted.

2. *In the Methods section of your revised manuscript, please amend lines 112-116 to match the description in lines 298-299 (Written informed consent for administration of any off-label medication was required by our institution).*

R: To avoid redundancy and misunderstanding, the sentence “Written informed consent for administration of any off-label medication was required by our institution” has been removed.

3. *In your Data Availability statement, you have not specified where the minimal data set underlying the results described in your manuscript can be found.*

R: Thank you for the remark. Minimal data set has been added as Supporting Information file.

4. *Please include captions for your Supporting Information files at the end of your manuscript, and update any in-text citations to match accordingly.*

R: File naming and in-text citations have been modified as per journal requirements.

**Response to Reviewer #1**

Major comments:

1. *The Authors should explain their definition of “severe” when defining bacterial co-infections*

R: Bacterial co-infection was considered “severe” when associated with life-threatening organ dysfunction (Singer et al. JAMA, 2016). Definition has been added in the text (Lines 105-106).

2. *It is not clear if patients who received anti-IL6 treatments were excluded or not, as in the paper 7% received tocilizumab; given that patients with immunosuppression were excluded, it does not seem that the Authors refer to previous treatment with anti-IL6 molecules for other condition; this issue should be clarified*

R: We thank the reviewer for the observation. Anti-IL6 treatment has been considered as an exclusion criterion for study enrollment as in our institutional protocol it was listed among the contraindications to anakinra administration. However, 18% (9/49) patients included in CG1 and 4.3% (2/47) patients included in CG2 received later during hospitalization, as part of the standard of treatment for COVID-19, accounting for 7.2% of the total population. In contrast, as shown in Table 1, patients who received anakinra did not receive anti-IL6 during the whole course of the hospitalization. The issue has been clarified in the text (Lines 121-124)

3. *Why did the Authors analyze two different cut-offs for severe respiratory failure (100 and 150)? Given the endpoints and the definition of ARDS I would use only the 150 cut-off.*

R: Thank you for the observation. We agree with the Reviewer, and we removed the 100mmHg cut-off.

4. *Was suPAR requested for all patients admitted with COVID-19 during the study period? If not, what are the characteristics of the patients for whom suPAR was and was not requested?*

R: Yes, once our laboratory implemented the suPAR assay, it was requested for every new COVID patient as part of the routine laboratory exams requested at admission.

5. *The selection of patients in the control groups does not appear to be clear. Were all patients with suPAR >6ng/mL included in the study, or were only patients used for the matching procedure considered? If so, how many patients presented with suPAR>6ng/mL, were not treated with ANK and were not included in the study? And what were the characteristics of these patients?*

R: Thank you for the observation. Matching was done by the authors of this study by directly looking for a suitable patient on the internal informatic system. For every patient treated with anakinra, all collectors followed the same matching procedure:

1. Searched for patients admitted for COVID-19 in the same time period (± 1 week).
2. Among patients admitted in the same time period, data collectors identified the ones with similar age (± 5 years), gender and vaccinal status. Those patients were then considered eligible for the study.
3. Screened for inclusion criteria.
4. Screened for exclusion criteria.

Hence, we did not collect data of all the other patients with baseline suPAR ≥ 6ng/ml, since the description of this population felt outside the scope of the present study.

More details about the matching procedure have been added in the text (Lines 112-120)

6. *In the statistical analysis section, more information should be provided regarding the matching procedure and the associated analysis (i.e., use of conditional logistic regression analysis, which is suggested for this kind of studies).*

R: As reported in the previous response, matching was done entirely by the authors of the study (E.R., F.S., F.C, F.S. and F.V.S.), actively looking for eligible patients within the hospital informatic system and, therefore, statistical analysis was introduced only upon data collection completion.

7. *The time from admission to administration of ANK should be described.*

R: We thank the Reviewer for the observation. Median time from admission to ANK administration was 1day (IQR: 1-2). We added this data to Table 1.

8. *The main outcome is disease progression at day 14, using a simplified version of the WHO-CPS; however, in Figure 1 the Authors evaluated progression to ARDS; I believe it would be more coherent with the main outcome to describe time to progression towards severe disease (which was defined as WHO-CPS 6-9), and possibly leave progression to ARDS as a secondary outcome; moreover, the definition of ARDS should be coherent throughout the text (Line 203-204: PF<150 and use of HFNC; line 216-217 PF <150 and use of HFNC/NIV or MV).*

R: We agree with the Reviewer: progression towards severe disease is indeed the main outcome, and progression towards ARDS is one the secondary outcomes. We clarified this in the Methods section (Lines 161-164) and, to avoid confusion, we inverted the naming of Figure 1 and Figure 2. As suggested by the reviewer, ARDS definition has been uniformed throughout the text.

9. *Results and table 3: where are the results of the matching cohort described? It seems from table 3 that 105 patients are included, i.e. 49+56. If a 1:1 matching was performed, the number of patients should be different.*

R: As reported in Table 1, matching cohort 1 and 2 (CG1 and CG2) were composed, respectively, by 49 and 48 patients. Unfortunately, to respect eligibility and inclusion criteria for controls, we were not able to reach exact 1:1 matching.

10. *The association between vaccine status and outcome is not statistically significant at univariate and multivariate analysis, so I would delete the phrase “Being vaccinated was in fact associated with a trend towards higher odds of disease progression, but this may be biased by the fact that vaccinated people were older and generally presented more risk factors for disease progression than non-vaccinated people (appendix, pg 4).”, while I would keep the following one (Line 272-275).*

R: We are grateful to the Reviewer for the observation. The phrase was removed from the text.

11. *How did you exclude patients considered for admission to ICU?*

R: We excluded patients considered for admission in ICU as they did not fulfil inclusion and exclusion criteria: in our center, all patients considered for COVID-ICU presented with severe respiratory failure with PaO2/FiO2 < 150mmHg requiring either high-flow oxygen therapy or NIV/MV.

12. *Figure 1: The difference between the two groups appears more significant in the first 24-48h. It seems that about 30% of patients in the control group progressed to ARDS in the first 24h, compared to 10% of patients in the ANK group, so it would be important to know the time of administration of ANK (see also the previous comment).*

R: We warmly thank the reviewer for this observation. It is correct, and it matches with the time of ANK administration (median: 1 day after admission). A dedicated sentence has been added in the Discussion section (Lines 306-310).

Minor comments:

13. *Line 92: 31rd should be changed to 31^st^*
14. *Line 194: “co-infections” should probably be changed to “superinfections”*
15. *Line 203: “defines” should be “defined”;*

R: The above sentences have been corrected.

16. *Secondary outcomes are “crude in-hospital mortality rate, length of hospitalization, development of severe respiratory failure with PaO2/FiO2 below 100 and 150 mmHg, and incidence of anakinra-related adverse events”; however, in the results section, supplementary oxygen and ICU admissions are also described. This should be added in the methods section.*

R: All secondary outcomes have been added in the Methods section (Lines 149-162).

17. *Lines 230-233: I would specify that patients analyzed did not receive anakinra (i.e: “performances of baseline suPAR and SCOPE score in predicting progression towards severe disease or death at day 14 from admission in patients non receiving ANK…”)*
18. *Line 320-321: baseline characteristics were not very similar between patients with suPAR >6 and <6, so I would just say that patients with suPAR >6 had similar characteristics.*

R: Thank you. We modified the sentences as suggested (Line 272 and 386).

**Response to Reviewer #2**

1. *As the study was retrospective, please make clear how written informed consent was received from participants. This is not clearly described and easy to follow.*

R: We thank the Reviewer for the observation. Along with (when necessary) asking for consent to off-label medications, in our center all COVID patients were asked for informed consent to use anonymized data for research purposes. This was a routine procedure in our hospital. This has been clarified in the methods section (Lines 126-127).

2. *Please describe in more detail the matching statistical procedure.*

R: Thank you for the observation. Matching was done by the authors of this study by directly looking for a suitable patient on the internal informatic system. For every patient treated with anakinra, all collectors followed the same matching procedure:

1. Searched for patients admitted for COVID-19 in the same time period (± 1 week).
2. Among patients admitted in the same time period, data collectors identified the ones with similar age (± 5 years), gender and vaccinal status. Those patients were then considered eligible for the study.
3. Screened for inclusion criteria.
4. Screened for exclusion criteria.

More details about the matching procedure have been added in the text (Lines 112-120).

3. *In the manuscript the authors describe that tocilizumab was an exclusion criterion but in Table 1 some participants have received tocilizumab. Please explain.*

R: We thank the reviewer for the observation. Anti-IL6 treatment has been considered as an exclusion criterion for study enrollment as in our institutional protocol it was listed among the contraindications to anakinra administration. However, 18% (9/49) patients included in CG1 and 4.3% (2/47) patients included in CG2 received later during hospitalization, as part of the standard of treatment for COVID-19, accounting for 7.2% of the total population. In contrast, as shown in Table 1, patients who received anakinra did not receive anti-IL6 during the whole course of the hospitalization. The issue has been clarified in the text (Lines 121-124).

4. *Table 1 and 2: it would be easier for the reader to provide here also p values of comparison with CG2 instead of supplement.*

R: We agree with the Reviewer. P-values obtained from the comparison of AG and CG2 have been moved from supplement to Table 1 and 2.

5. *Incidence of bacterial co-infection was similar: it would be very interesting to provide these data.*

R: The seven co-infections reported in the study were all presumptive bacterial pneumonia complicating COVID-19. Clinical suspicion raised mainly from CT-scan presentation and elevation in the polymorphonucleate count ad admission. All patients were prescribed wide-spectrum empirical antimicrobial therapy. Unfortunately, none of the reported co-infections resulted positive to blood or sputum cultures.

6. *Lines 288-299: here the authors discuss something very important, namely the Hawthorne effect. It is very important to have real world data and see how a strategy developed in the context of a clinical trial finds applicability and is easily implemented in everyday routine. This is of great value in real world data studies such as this one, which is the first after the two clinical trials for suPAR guided anakinra.*

R: We warmly thank the Reviewer for this comment.

We are thankful for all the issues highlighted by reviewers. Hence, we believe that reviewer’s observations substantially improved the quality of our work.

Kind regards,

Francesco Vladimiro Segala
