# Supplementary material for: Anakinra in hospitalized COVID-19 patients guided by baseline soluble urokinase plasminogen receptor plasma levels: A real world, retrospective cohort study
Source: PLoS One. 2023 Apr 4;18(4):e0273202. doi: 10.1371/journal.pone.0273202 (PMC10072376; doi:10.1371/journal.pone.0273202)
Supplement: Attachment — Submitted filename: Response to Reviewers v2.docx [file pone.0273202.s008.docx]

Dear PLOS-One Editor,

please find attached a point-by-point response to the Reviewer’s comments.

**Response to Reviewer #1**

Major comments:

1. *For the analysis of the primary outcome, the matching between the CG1 and the anakinra group should be 1:1 to respect the assumption of a mahed-cohort study; in order to reach a 1:1 matching, therefore, some patients of the anakinra group who do not have an exact matching should be excluded from the analysis*

R: Thank you for your considerable observation. In order to improve the internal validity of our study, we performed the analysis with a more rigorous methodology. Indeed, although the two cohorts initially appeared to be well-balanced with respect to important baseline characteristics, subtle imbalance may still be present if only looking at the *p*-value due to a small sample size effect. Thus, we evaluated the standardized mean differences (SMD) of the covariates between the two groups; in this way, we found that several covariates were actually differently distributed. For this reason, we went further by creating a propensity score (PS) of receiving anakinra to avoid unbalanced distribution of variables. After that, we use the PS to estimate the inverse probability of treatment weighting (IPTW), conducting the analysis adjusting for these weights. By such a method, we were able to use all patients, regardless of exactly matching 1:1, by assigning each patient a weight [1].

2. *The Authors state that ordinal regression analysis was used to explore risk factors associated with progression to severe disease or death; however, if the ordinal scale ranging from 6 to 10 was used as dependent variable, some of the categories would have 0 patients (especially in the anakinra group, where there are 2 patients in the “severe disease” group, ranging from 6 to 9); it would be more adequate to perform a binomial logistic regression analysis using as dependent variable progression to severe disease or death vs. no progression to severe disease or death)*

R: Thank you for the observation. For ordinal logistic regression, we used a simplified version of the simplified version of the 11-point World Health Organization Clinical Progression Scale, ranging from 1 (uninfected/ambulatory disease) to 4 (death). Severe disease (WHO-CPS score 6-9) are categorized with a score of 3 in the simplified version (lines 129-134, methods section), thus all patients are categorized. Unfortunately, due to the retrospective nature of our data, we were not able to use the full WHO-CPS scale. We agree with the reviewer that this represents a limitation, and we added a paragraph to highlight this in the dedicated section (lines 407-409). However, even using a simplified scale, with the aim to be more comparable with the SAVE-MORE results, the authors of this work consider ordinal regression analysis as a good tool to represent our results.

3. *Given the number of patients with the outcome of interest (14 patients, according to table 1), the number of variables used in the multivariate analysis appears too high, according to the commonly used rule-of-thumb; moreover, it is not clear how the variables were chosen to be included in the multivariate analysis (as, for example, BMI and sex were not associated with the outcome in the univariate analysis); it would be more prudent to reduce the number of variables included in the multivariate analysis, possibly by selecting variable with significant p-values at univariable analysis.*

R: Thank you very much for the precious observation. As stated in the response to the first comment, we modified the analysis using a propensity score of receiving anakinra along with the IPTW. We selected the variables to include in the PS by looking if the standardized mean differences of the covariates between the two groups were > 0.1 since this cutoff is generally considered a meaningful difference. Concerning the variables to include in the propensity-adjusted multiple logistic regression, we follow the rule of thumb as suggested to improve the validity of the results. Indeed, we included only 3 variables: anakinra use, age in years, and PaO_2_/FiO_2_ ratio. Those variables were selected by investigators’ consensus and clinical importance. We used these variables due to their profound impact on the outcome in order to evaluate the role of anakinra independently of them. Moreover, even if some covariates were not perfectly balanced in the PS (WBC, CRP, and PaO_2_/FiO_2_ ratio), we included only the PaO_2_/FiO_2_ ratio to further adjust for it; with respect to the other two variables, we avoided including them due to their SD < 0.25 not deeply influencing the overall balance, and to limit the number of predictors. We preferred not to use variables below a certain cutoff of *p* due to some caveats associated with this method, similar to stepwise selection strategies [2]. Doing so, we argued that our model is more explanatory respecting its original purpose and avoiding possible misleading results due to possible false-positive *p*.

4. *The statistical methods for the primary outcome should then be better specified in the “Statistical analysis” section.*

R: Thank you for your observation. We do agree and we have re-written the statistical analysis section (lines 140-178).

Minor comments:

5. *I would add the word “manually” in line 111 (...patients included in both control groups were manually matched…)*

R: We added the word “*manually”* as suggested.
